# Subnormothermic Ex Vivo Lung Perfusion Temperature Improves Graft Preservation in Lung Transplantation

**DOI:** 10.3390/cells10040748

**Published:** 2021-03-29

**Authors:** Stephan Arni, Tatsuo Maeyashiki, Necati Citak, Isabelle Opitz, Ilhan Inci

**Affiliations:** Department of Thoracic Surgery, University Hospital Zurich, Rämistrasse 100, 8091 Zurich, Switzerland; stephan.arni@usz.ch (S.A.); tmaeya@juntendo.ac.jp (T.M.); necomomus@gmail.com (N.C.); Isabelle.Schmitt-Opitz@usz.ch (I.O.)

**Keywords:** ex vivo lung perfusion, subnormothermic perfusion, lung transplantation

## Abstract

Normothermic machine perfusion is clinically used to assess the quality of marginal donor lungs. Although subnormothermic temperatures have proven beneficial for other solid organ transplants, subnormothermia-related benefits of ex vivo lung perfusion (EVLP) still need to be investigated. **Material and Methods:** In a rat model, we evaluated the effects of 28 °C temperature on 4-h EVLPs with subsequent left lung transplantation. The recipients were observed for 2 h postoperatively. Lung physiology data were recorded and metabolic parameters were assessed. **Results:** During the 4-h subnormothermic EVLP, the lung oxygenation was significantly higher (*p* < 0.001), pulmonary vascular resistance (PVR) lower and dynamic compliance (Cdyn) higher when compared to the 37 °C EVLP. During an end-of-EVLP stress test, we recorded significantly higher flow (*p* < 0.05), lower PVR (*p* < 0.05) and higher Cdyn (*p* < 0.01) in the 28 °C group when compared to the 37 °C group. After the left lung transplantation, Cdyn and oxygenation improved in the 28 °C group, which were comparable to the 37 °C group. Chemokines RANTES, MIP-3α, MIP-1α MCP-1 GRO/KC and pro-inflammatory mediators GM-CSF, G-CSF and TNFα were significantly lower after the 28 °C EVLP and remained low in the plasma of the recipient rats after transplantation. The lungs of the 28 °C group showed significantly lowered myeloperoxidase activity and lowered levels of TNFα and IL-1β. **Conclusions:** Compared to the normothermic perfusion, the 28 °C EVLP improved Cdyn and PVR and reduced both the release of pro-inflammatory cytokines and myeloperoxidase activity in lung tissue. These observations were also observed after the left lung transplantation in the subnormothermic group. The 28 °C EVLP significantly improved biochemical, physiological and inflammatory parameters in lung donors.

## 1. Introduction

The ex vivo lung perfusion (EVLP) was developed in order to increase lung usage by re-evaluating, treating, and repairing questionable donor lungs [1]. EVLP provides a way to improve the use of questionable donor lungs by allowing physiological and radiological evaluation of explanted donor lungs that are considered “marginal”. The EVLP gives the clinicians a second opportunity to decide whether to perform the lung transplantation or not, instead of prematurely turning down an organ that appears questionable according to standard clinical criteria alone.

Normothermic perfusion has already been studied and proven to enable organ viability assessment before transplantation [2]. However, in this study, we investigated the effect of subnormothermic perfusion temperature as we suspect it to be one of the key factors for damaged graft improvement. We evaluated the effect of subnormothermic perfusion temperature during 4 h of EVLP at 28 °C and then performed left lung transplantation [3,4] in order to improve organ preservation through the cytoprotective benefits of reduced cellular metabolism. 

## 2. Materials and Methods

### 2.1. Animals

The Kanton Zurich Veterinarian committee approved this study (ZH228/16). We selected male Sprague Dawley rats weighing 280–360 g (Janvier Labs, France) for these experiments. The rats were maintained in a pathogen free area and received adequate care according to “Guide for the Care and Use of Laboratory Animals: Eighth Edition” [5].

### 2.2. Surgical Technique and Rat Lung Model for EVLP

The rats were anaesthetized with a mixture of oxygen and isoflurane and underwent tracheotomy and mechanical ventilation with a rodent ventilator (Harvard Apparatus, Inc., Model Ventelite, Hugstetten, Germany). A tidal volume of 10 mL/kg was applied with a respiratory rate of 60 breaths/min, and a positive end-expiratory pressure (PEEP) of 3 cmH_2_O [3,6]. Following laparotomy and sternotomy, the rats were heparinized with 300 IU intravenous heparin via the inferior vena cava. We performed a 2–3 mm incision on the anterior surface of the right ventricular outflow tract and placed the cannula in the incision and into the main pulmonary artery and secured it with a silk suture. Next, we made a 4–5 mm incision on the apex of the left ventricule and placed a cannula into this incision and pushed it into the left atrium through the mitral valve. After the cannulation, we opened the inferior vena cava and performed anterograde flush with Perfadex^®^ at a perfusion pressure of 20 cmH_2_O. The trachea was clamped and the lungs inflated with a sustained airway pressure of 15 cmH_2_O. The harvested heart-lung block was placed in a plastic bag containing 15 mL of Perfadex^®^, double bagged, then stored for a 1 h of cold ischemic time. 

#### 2.2.1. Physiological Variables and Clinical Biochemistry Parameters

We recorded all the respiratory parameters with a dedicated software (PULMODYN^®^ software, Hugo Sachs Elektronik Harvard Apparatus, Hugstetten, Germany) under positive pressure ventilation and monitored pulmonary arterial pressure (PAP), peak airway pressure, and airway flow during the 4 h of EVLP. The dynamic lung compliance (Cdyn) and the pulmonary vascular resistance (PVR) were also analyzed. 

#### 2.2.2. Normothermic and Subnormothermic EVLP Treatment Groups

**Figure 1:**
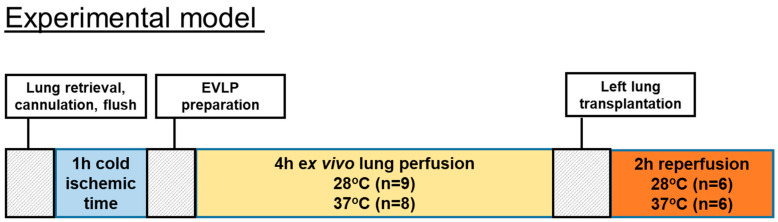
Timeline of the animal study.

EVLP was started with 125 mL of Steen solution (Steen solution, Xvivo Perfusion, Göteborg, Sweden) containing heparin 300 IU, antibiotic (50 mg meropenem), and methylprednisolone (50 mg). Before the start of the EVLP procedure, we oxygenated the Steen solution at a 30 mL/min flow for 15 min at 20 °C through an oxygenator column with 2 L per minute flow of 100% oxygen. We started to perfuse lungs at 20 °C and with 10% of the targeted flow that was calculated as the 20% of a 250 g weight rat cardiac output. We then gradually raised temperatures and flow during the first hour toward a 100% target flow. The targeted sub-normothermic (28 °C) and normothermic (37 °C) perfusion temperatures were reached after 50 min and kept onwards during the 4 h of EVLP. The IPL-2 system (IPL-2, Hugo Sachs Elektronik Harvard Apparatus, Germany) was set during the 4 h of EVLP to maintain the PAP below 15 cmH_2_O by automatically adjusting the flow. We used a fixed tidal volume of 5 mL/kg at a respiratory rate of 30 breaths/min with a PEEP of 3 cmH_2_O. At the end of the 4 h of EVLP, an additional 5 min stress test was performed where the flow was enabled to increase until reaching a maximum PAP of 15 cmH_2_O. The number of performed EVLP was n = 9 for the subnormothermic group and n = 8 for the normothermic group. The timeline of the animal study is shown in Figure 1. 

The number of EVLPs was n = 9 for the subnormothermic group and n = 8 for the normothermic group. Six transplantations were performed in each of the normothermic and subnormothermic groups. After left lung transplantation, the rats’ lungs from both groups were reperfused and hemodynamic data were collected during a 2-h postoperative period. Finally, blood oxygenation parameters were recorded and the plasma and the pulmonary tissues were collected.

### 2.3. Biochemical Measurements

The lungs were evaluated hourly. During this evaluation phase, the lung was ventilated with 100% O_2_ for 5 min, and the perfusate was sampled for pulmonary oxygenation, pH, concentrations of potassium, calcium, sodium, glucose, lactate and the partial oxygen pressure with the Epoc^®^ blood analysis system (Epoc^®^ Blood Analysis System, Siemens Healthineers, Erlangen, Germany). The change in PaO_2_ (ΔPaO_2_) was calculated as the partial pulmonary venous PO_2_—pulmonary arterial PO_2_.

#### 2.3.1. Rat Lung Transplantation Procedure

Recipient animals were anaesthetized with a mixture of oxygen and isoflurane, and intubated. Anaesthesia was maintained with 1–2% isoflurane during the operation and reperfusion period. For measuring the airway pressure during the procedure, a three-way stopcock was inserted between the intratracheal tube and the ventilator circuit and was connected to a pressure transducer. A left thoracotomy was performed through the fifth intercostal space. The left lung was mobilized by dividing the pulmonary ligament. The hilum of the left lung was dissected, and the pulmonary artery, pulmonary vein and the left main bronchus were isolated. All three structures were clamped by using microsurgical aneurysm clamps. They were incised on their anterior aspect, and the cuffs of the donor lung were placed into the equivalent recipient structures and fixed with a 6–0 polypropylene suture. The transplanted lung was inflated and pulmonary vein and arterial clamps were released, respectively. The thoracotomy was closed loosely. The recipient animal got ventilated (with 2–3% (*v*/*v*) isoflurane in O_2_, a tidal volume of 10 mL/kg at 75 breaths/min, and a PEEP of 3 cmH_2_O) for 2 h [3]. During these 2 h, we analyzed the left lung function. After 2 h, oxygenation of the graft was evaluated by sampling the blood directly from the pulmonary vein of the transplanted lung by means of heparinized needle aspiration inserted distally to the anastomotic cuff and, finally, the heart-lung block got explanted. For further biochemical analysis, an aliquot of the left lungs was flash frozen in liquid nitrogen. All samples were stored at −80 °C until further examinations. Six transplantations were performed in the normothermic and subnormothermic groups. The timeline of the animal study is shown in Figure 1.

#### 2.3.2. Cytokines, Chemokines and Mediators of Tissue Repair

We assayed 50 µL of perfusate or plasma for cytokines and chemokines levels with a mouse cytokine/chemokine panel Bio-Plex Pro Mouse Cytokine 23-plex (Bio-Rad Laboratories, Hercules, CA, USA) according to the manufacturer’s instructions. The Steen perfusate collected after 4 h of EVLP was flash-frozen in liquid nitrogen and stored at −80 °C. After 2 h of reperfusion, the whole blood was collected in ethylenediaminetetraacetic acid-treated tubes (BD Microtainer tubes cat 365975). We centrifuged the whole blood for 10 min at 4 °C and 3300 rpm to separate blood cells from plasma. The supernatant was collected, transferred in a clean polypropylene tube and centrifuged for 10 min at 4 °C and 4000 rpm. The collected supernatant transferred in a clean polypropylene tube was flash-frozen in liquid nitrogen and stored at –80 °C until use. Tissue lysates extracted from the powdered lung tissue were also analysed by ELISAs for rat IL-6 (DY-506, R&D Duoset Systems, Minneapolis, MN, USA), for rat TNF-α (DY-510, R&D Duoset Systems, Minneapolis, MN, USA) and for rat IL-1β (DY-501, R&D Duoset Systems, Minneapolis, MN, USA) according to manufacturer’s instructions. The lung tissue lysates were prepared by glass-on-glass pestle homogenisation and at a ratio of 1.25 mg powdered lung tissue per ml (*w*/*v*) of ice cold DPBS containing a protease cocktail inhibitor (complete EDTA-free protease inhibitor tablets, Roche, Rotkreuz, Switzerland). We centrifuged the lysates for 5 min at 4 °C and 14,000 rpm to separate cleared supernatant from insoluble cell debris. The supernatant were loaded in duplicate on ELISA plates.

#### 2.3.3. Estimates of ATP Content, Myeloperoxidase Activity and Carbonyl Protein Content in Lung Tissues and Histologic Studies in Lung Sections

Frozen lung tissues were crushed on dry ice and the powdered tissues were used to prepare the lysates. ATP content was determined from 25 mg of powdered lung tissue and was homogenized in 0.5 mL of 0.5% trichloroacetic acid and centrifuged at 8000 rpm for 2 min at 4 °C. The supernatant was isolated and 10 µL of 0.002% xylenol blue and 10× concentrated Tris-acetate buffer was used to neutralize pH to 7.4. We used an ATP assay kit (Enliten, Promega, Madison, WI, USA) to estimate the ATP concentration in the supernatant by measuring in the luminescence channel of a Cytation 5 plate reader (BioTek Instruments, Inc., Winooski, VT, USA). The Pierce microBCA kit was used as a protein assay with bovine serum albumin as standard to determine protein concentration in the supernatant according to the manufacturer’s instructions (Thermo Scientific, Rockford, IL, USA). The results were expressed in nanomolar ATP per milligram of proteins. Tissue lysates extracted from the powdered lung tissue were also analyzed using (1) a myeloperoxidase (MPO) activity assay (OxiSelect™ myeloperoxidase chlorination activity assay, Cell Biolabs, San Diego, CA, USA) and (2) an ELISA-based carbonylated proteins assay (OxiSelect™ carbonyl protein ELISA assay, Cell Biolabs, San Diego, CA, USA) according to manufacturer’s instructions. Tissue samples were collected, fixed in formalin and embedded in paraffin. Tissue sections were stained with haematoxylin and eosin for microscopic assessment. Lung injuries were assessed according to a previously published scoring system [7].

### 2.4. Statistical Method

Results are expressed as mean and standard deviation (SD). For cytokine analysis the median and interquartile range (IQR) were used as measures of central tendency and dispersion, respectively. A nonparametric Mann–Whitney U-test was used for non-continuous data. Data with a time component was compared using 2-way analysis of variance (ANOVA). Statistical analysis was performed with GraphPad Prism version 8 software (GraphPad Software, Inc., La Jolla, CA, USA). Differences were considered significant at *p* < 0.05.

## 3. Results

### 3.1. Lung Physiology during EVLP and Transplantation

**Figure 2:**
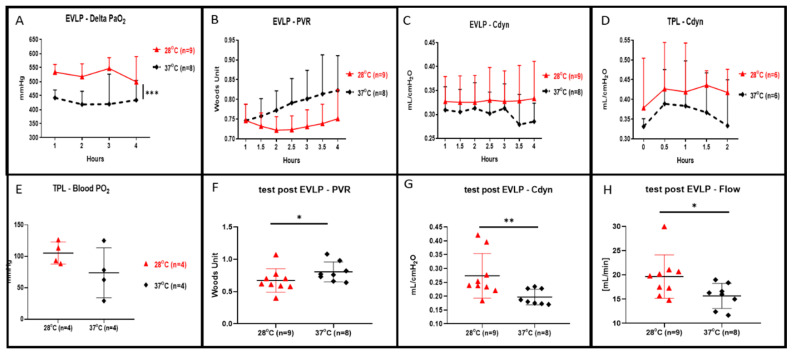
Lung oxygenation, pulmonary vascular resistance (PVR), dynamic compliance (Cdyn) and flow during 4 h of ex vivo lung perfusion (EVLP) and after 2 h of transplantation. (**A**) Perfusate oxygenation was significantly improved in EVLP done at 28 °C when compared to EVLP done at 37 °C temperature (*** *p <* 0.001). (**B**) PVR during the 28 °C EVLP was lower but not statistically different from the EVLP done at 37 °C. (**C**) Cdyn during the 28 °C EVLP was higher but were not statistically different from the EVLP done at 37 °C. (**D**) During 2 h of post EVLP transplantation reperfusion time, the compliance measured from the rat allocated to the 28 °C EVLP group was higher but not statistically different from the compliance of transplanted lungs originating from the EVLP done at 37 °C. (**E**) At the end of the 2 h post-transplantation reperfusion, the oxygenation from the pulmonary vein was higher in the lungs allocated from the 28 °C EVLP group but was not statistically different when compared to the control group. (**F**) In the 28 °C EVLP group and during the 5 min end of EVLP stress test we recorded a significant lower PVR (* *p <* 0.05) and (**G**) a significantly higher Cdyn (* *p <* 0.05, ** *p <* 0.01) and (**H**) a significantly higher flow (* *p <* 0.05) when compared to the normothermic group.

During the 4 h of EVLP, we recorded a statistically higher oxygenation in the EVLP perfusion done at 28 °C in comparison to the control (shown in Figure 2A, *p* < 0.001). At a 28 °C subnormothermic temperature and during 4 h of EVLP, we also observed both decreased lung PVR (shown in Figure 2B) and increased lung Cdyn (shown in Figure 2C) but those values were not statistically different when compared to normothermic EVLP. At the end of the 4 h at 28 °C EVLP and after the 5 min stress test, we recorded a significant increase in flow (shown in Figure 2H, *p* < 0.05), a significantly lower PVR (shown in Figure 2F, *p* < 0.05) and significantly higher Cdyn (shown in Figure 2G, *p* < 0.01) compared to the EVLP done at normothermia. After 4 h of EVLP, left lungs were transplanted into recipient rats and perfused for 2 h under anesthesia. The lung Cdyn of the transplanted rats allocated to the 28 °C EVLP group were higher, however, those differences were not statistically different from the Cdyn value of the control EVLP (shown in Figure 2D). Moreover, the lungs of the 28 °C EVLP group transplanted into the recipient rats presented enhanced oxygenation, however, did not differ significantly from the lungs of the control EVLP condition transplanted into recipient rats (shown in Figure 2E).

### 3.2. Clinical Biochemistry in EVLP Perfusate and in Transplanted Recipients

**Figure 3:**
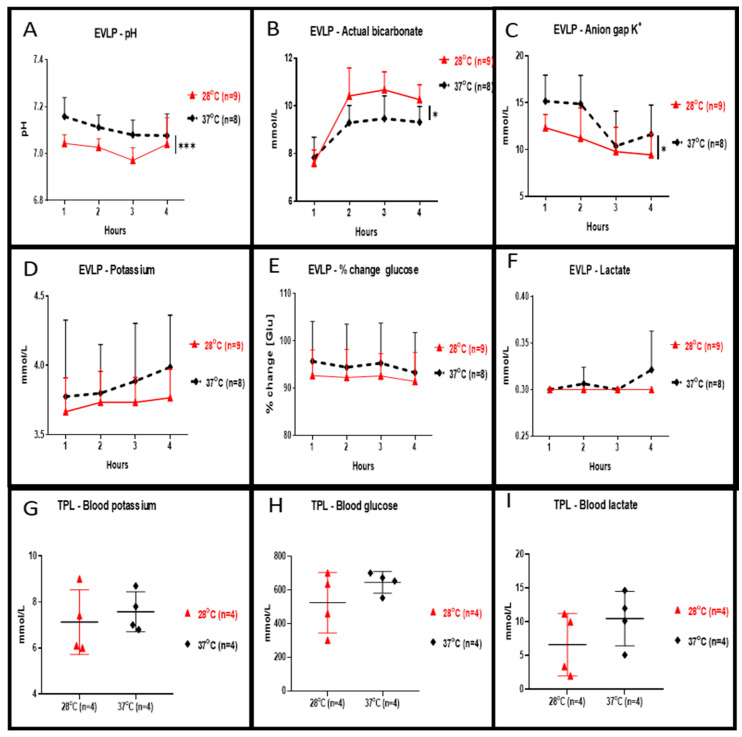
Clinical biochemistry during EVLP and after left lung transplantation. During EVLP done at 28 °C we recorded: (**A**) a significantly lower pH (*** *p* < 0.001), and (**B**) bicarbonate levels significantly higher (* *p* < 0.05), and (**C**) significantly lower anion gap K+ values (* *p* < 0.05) when compared to EVLP done at 37 °C. The potassium concentration of the perfusate (**D**) and in the plasma (**G**) were lower at 28 °C but the concentration was not statistically different when compared to the 37 °C groups. (**E**) The glucose concentration in the 28 °C EVLP perfusate and (**H**) in the plasma of transplanted rats from the 28 °C group was not significantly lower when compared to experiments done at 37 °C. (**F**) In the 28 °C EVLP perfusate and (**I**) in the plasma of transplanted rats of the 28 °C group, the lactate concentration was not significantly lower when compared to experiments done at 37 °C. TPL: transplantation.

Perfusate concentration of potassium (shown in Figure 3D), percentage changes of glucose (shown in Figure 3E) and lactate (shown in Figure 3F) during the 4 h of EVLP at 28 °C were slightly reduced but were not statistically different when compared to 37 °C. At 28 °C, perfusate values of pH (shown in Figure 3A, *p <* 0.001) and of anion gap K^+^ (shown in Figure 3C, *p <* 0.05) were significantly lower whereas values of bicarbonate were significantly higher (shown in Figure 3B, *p <* 0.05). Plasma levels of potassium (shown in Figure 3G), glucose (shown in Figure 3H) and lactate (shown in Figure 3I) at the end of the 2 h reperfusion in transplanted rats allocated from in 28 °C EVLP group were reduced but were not statistically different when compared to the control group.

### 3.3. EVLP Perfusate, Plasma and Tissue Cytokines, Chemokines and Tissue Repair Mediators

In Table 1, the cytokines, chemokines, growth factors and mediators of tissue repair levels are shown; (1) in the perfusate after 4 h of EVLP, (2) in the plasma or (3) in the lung tissue of recipient rats 2 h after the left lung transplantation. The pro-inflammatory mediator TNFα was significantly decreased in the perfusate after 4 h of EVLP at 28 °C (*p* < 0.001). Some chemokines mediators of leukocytes trafficking such as RANTES (*p* < 0.05), MIP3α (*p* < 0.05), MIP1α (*p* < 0.05), MCP-1 (*p* < 0.001) and GRO/KC (*p* < 0.001) and cytokines such as GM-CSF (*p* < 0.05) and G-CSF (*p* < 0.05) were also significantly lower in the perfusate after 4 h EVLP at 28 °C. Some pro-inflammatory cytokines such as IL-6, IL-7 were also lower in the perfusate after 4 h of EVLP at 28 °C although those cytokine reductions were not statistically different compared to that of EVLP done at 37 °C.

In the plasma of transplanted rats with EVLP done at 28 °C, we also recorded lower levels in some pro-inflammatory mediators such as TNFα, or of chemokines mediators of leukocytes trafficking such as MIP3α, MIP1α, GRO/KC and the cytokine GM-CSF although those differences were not statistically different from that of the plasma of rats allocated in the 37 °C group. In the tissue of transplanted rats with EVLP done at 28 °C, we recorded significantly lower levels in the pro-inflammatory mediators TNFα (*p* < 0.05) and IL1-β (*p* < 0.05). We recorded lower levels of the cytokine IL-6 but the difference was not statistically different from that of the tissue of rats allocated in the 37 °C group. Some chemokines such as MCP-1 and some cytokines such as IL1-β, IL-6 and the wound healing and tissue repair mediator VEGF were lower in the perfusate after 4 h of EVLP at 28 °C and in the plasma of the 28 °C group of transplanted rats, although those differences were not statistically significant compared to the perfusate or the plasma of the 37 °C group.

Interestingly, IFNγ and some cytokines such as IL-12(p70), IL-13, IL-17A, IL-18A or some anti-inflammatory cytokines such as IL-10 and IL-4, were higher in the plasma of transplanted rats after the 4 h of EVLP done at 28 °C although those differences were not statistically significantly from that of the plasma of transplanted lung collected from EVLP done at 37 °C.

### 3.4. Lung Tissue Content in MPO, ATP and Carbonyl Proteins, and Histological Studies of Lung Sections

**Figure 4:**
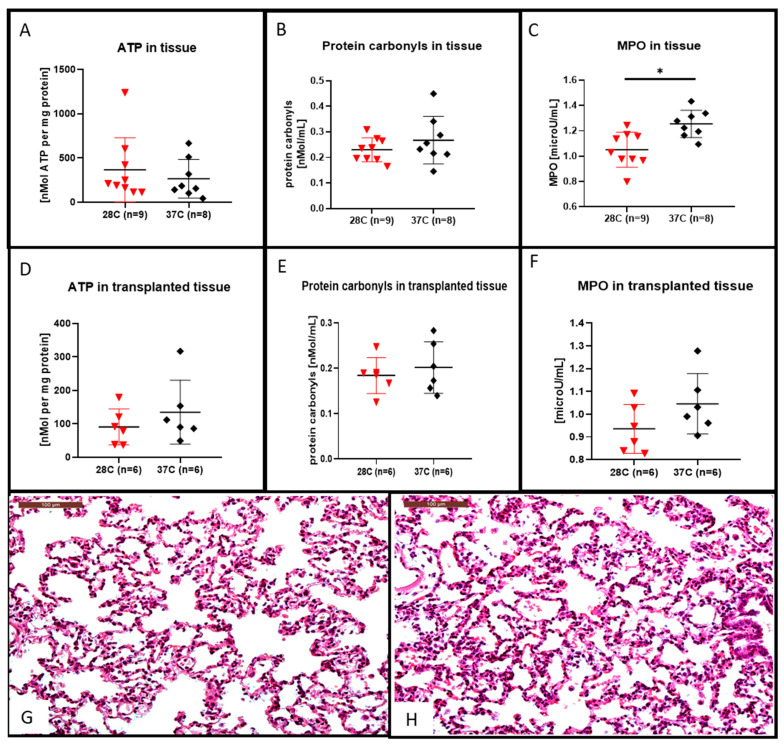
Hematoxylin and eosin stained lung sections, ATP, protein carbonyl contents and myeloperoxidase (MPO) measurement in lung tissues. After the 4 h EVLP at 28 °C, the ATP content was increased (**A**), the protein carbonyl content was decreased, (**B**) although those differences were not significant when compared to the 37 °C EVLP. The MPO content recovered after 4 h of EVLP at 28 °C was significantly lower than the control at 37 °C, ((**C**) * *p <* 0.05). In the transplanted lung tissue of the 28 °C group, the ATP content (**D**) and the protein carbonyl content (**E**) were both decreased, although those differences were not significant when compared to the 37 °C group. In (**F**), the MPO content recovered 2 h after transplantation in the 28 °C group was lower, although those differences were not statistically different from the control at 37 °C. Representative hematoxylin and eosin stained sections for assessment of lung injuries in the subnormothermic group (**G**) and the normothermic group (**H**). Sections are shown at X200 magnification at the end of the 2 h transplantation period. The scale bar indicates 100 µm length.

After the 4 h of EVLP at 28 °C we observed that the lung tissue ATP content was higher (shown in Figure 4A) and the protein carbonyl tissue content was lower (shown in Figure 4B) although they were not statistically different from that of the lung tissue perfused at 37 °C. This decrease in protein carbonyl tissue content after EVLP done at 28 °C was also observed in the lung tissue after 2 h of transplantation (shown in Figure 4E). For the transplanted lung tissue, the ATP content was rather higher in the 37 °C group, although not statistically different from that of the lung tissue content of ATP after EVLP done at 28 °C (shown in Figure 4D).

After the 4 h of EVLP, the lung tissue MPO content were significantly lower at 28 °C (shown in Figure 4C, *p <* 0.05) when compared to their amount obtained after 4 h of EVLP done at 37 °C. This decrease in MPO content in the group of lungs processed with a 28 °C EVLP was also observed after a post-EVLP reperfusion period of 2 h in the left lung, although this decrease was not statistically different from that of the MPO lung tissue content in the group of lungs perfused at 37 °C (shown in Figure 4F). We did not observed significant differences in the microscopic assessment of lung injuries (i.e., inflammatory cell infiltration or alveolar haemorrhage) at the end of the 2-h transplantation period in either subnormothermic (shown in Figure 4G) or the normothermic (shown in Figure 4H) lung tissues.

## 4. Discussion

Lung machine perfusion assessment at normothermic temperature is performed before transplantation. The subnormothermic machine perfusion settings are already in clinical use for other solid organs [8,9,10,11,12,13]. We hypothesized that the use of subnormothermic temperature in a rat EVLP platform may positively impact physiological, biochemical and inflammatory parameters in lung donors. The assessment of donor lung quality during EVLP relies mainly on the monitoring of airway pressure, pulmonary artery pressure, and oxygen concentration. The perfusate oxygenation of the subnormothermic EVLP condition was significantly improved but marginally improved in the transplanted lungs. An obvious explanation is that gas solubility typically decreases as temperature increases as would apply here for the 28 °C versus the 37 °C temperature. Following the 5 min stress test performed at the end of the subnormothermic EVLP, we recorded a significantly higher flow, lower PVR and higher Cdyn versus the normothermic EVLP perfusion. In this setting and despite the deleterious effect of cold on both the sensitive lung parenchyma surfactant biophysical properties (surface tension) and of the blood vessels (vasoconstriction), the physiological data of the subnormothermic condition (PVR, Cdyn, delta PO_2_) point towards a non-deleterious and protective effect ending in a better physiological state of the donor graft after 4 h of EVLP and after the 2 h following transplantation compared to the normothermic condition. As both the liver and kidneys are absent from acellular organ machine perfusion protocol, the general observation is that the circulating concentration of potassium, sodium, and lactate increases as the pH drops. The potassium levels in a perfusate may also be used as an indirect proxy for cell integrity [14] and cell death [15] and potassium concentrations were always lower during the 28 °C EVLP and in the plasma of the transplanted rats. Interestingly, the pH was significantly lower in the EVLP done at 28 °C. This reduced pH during the 28 °C EVLP was recorded showing a significantly higher bicarbonate concentration and significantly lower anion gap K^+^ value when compared to EVLP done at 37 °C. Here, the non-significant increased lactate concentration during EVLP at 37 °C may have participated to a higher anion gap K^+^ through lactic acidosis. Lactic acidosis results from the excess formation of lactate, which occurs during states of anaerobic metabolism. The anaerobic glycolysis is the process by which the normal pathway of glycolysis is routed to produce lactate and this metabolic waste product will accumulate over time [16]. Lactate accumulation occurs at times when energy is required in the absence of oxygen and the preoperative serum lactate levels is a strong independent predictor of worse outcomes in patients undergoing urgent heart transplantation on short-term mechanical circulatory support [17]. Nonetheless, for patients who underwent lung transplantation after EVLP their outcomes were good despite increased lactate during EVLP [16]. In our experiments, the lactate concentrations at 28 °C in transplanted rat plasma or during EVLP were lower but not significantly different from the condition at 37 °C. We also recorded a higher, yet not significantly different decrease/consumption of glucose in both the EVLP and plasma of the subnormothermic group when compared to the normothermic group. Since there are no compensatory mechanisms in this EVLP setting, the decreased lactate levels or reduced glucose consumption levels may indicate respectively, aerobic glycolysis in the 28 °C group and increased anaerobic glycolysis in the 37 °C group. During EVLP, those changes are mainly attributable to the pneumocyte metabolism whereas during transplantation they are the consequence of an accumulation from the whole animal metabolism and occurs at times when energy is required in the absence of oxygen. All these clinical chemistry data for potassium, anion gap K^+^ but also lactate and glucose levels in the EVLP perfusate or the plasma of transplanted animals point towards a beneficial effect of the EVLP done at 28 °C subnormothermic temperature compared to the 37 °C group.

Several biochemical parameters of the lung tissue were also improved by lowering the machine perfusion temperature. The ischemia-reperfusion (I/R) lesions occur as a result of a sequence of events such as the loss of blood flow, a period of ischemia and cold exposure, and a period of reperfusion [18]. The MPO enzymes are released into the extracellular fluid because of oxidative stress or during an inflammatory response. MPO activity, released from neutrophils, was significantly reduced under subnormothermic temperature conditions. Other I/R damages could also be reduced in the subnormothermic EVLP condition, although not significantly, such as the depletion of ATP stocks in mitochondria leading to altered permeability of the mitochondrial membrane and depolarizing their membrane potential [19]. During the subnormothermic temperatures perfusion, the reactive oxygen species (ROS) release, which is an injury factor triggered during the hypothermic period [20] in both liver [21,22] and lung [23] transplantation settings, has also improved through a reduced but not significantly different concentration of carbonyl protein content in lung tissue. As protein carbonylation is a type of protein oxidation that can be promoted by ROS [24,25] one hypothesis would be that subnormothermic temperature perfusion would decrease mitochondrial ROS generation and mitochondrial transition pore opening and permeability, ultimately leading to a lessened release of damage-associated molecular patterns (DAMPS) [26]. DAMPS are associated with a subsequent elevation of tissue damage and inflammation. We recorded a significantly reduced amount of the pro-inflammatory cytokines, chemokines and growth factors selected to characterize the 28 °C EVLP perfusate (TNFα, MCP-1, RANTES, MIP3α, MIP1α, GRO/KC, GM-CSF and G-CSF), or expressed in the lung tissue (IL1-β, TNFα) following the 2 h post-transplantation reperfusion period and with similar but not significantly decreased levels of these analytes in the plasma when compared to the 37 °C group. Interestingly, some anti-inflammatory cytokines such as IL-10 and IL- 4, were increased in the plasma of transplanted rats after the 4 h of EVLP done at 28 °C although those differences were not statistically significantly from that of the plasma of transplanted lung collected from EVLP done at 37 °C. After EVLP-induced I/R injury, some pro-inflammmatory cytokines were associated with a later higher rates of primary graft dysfunction (PGD) when implanted into a recipient [27]. Nonetheless, and regardless of the PGD score, previous clinical studies have shown that, IL-6, IL-8, and IL-10 increase in the plasma of patients early after reperfusion [28,29].

Recently, Gloria et al. presented evidences after a 2-h reperfusion period in recipient rats, that lung grafts treated pre-transplantation without EVLP but exposed to a 4-h period of cold ischemic time have a worst outcome than their three experimental groups of undamaged lungs treated before transplantation with EVLP temperature set at either 25 °C, 30 °C or 37 °C [30]. Instead of undamaged lungs, and in order to mimic as close as possible the clinical situation, our study used lungs damaged by exposure to a 1-h cold storage time (cold ischemia) before EVLP. We present evidences that subnormothermic EVLP in a clinically relevant rat donor lung model has strong beneficial potential for both physiological parameters and for the attenuation of I/R injury when compared to the normothermic temperature. We hypothesize that subnormothermic temperature extended the preservation of the graft by two mechanisms; (1) an attenuated intensity of the inflammatory cytokine burst during EVLP and consequently a reduced release of oxidative stress related enzymes active into the extracellular fluid or/and (2) an higher mitochondrial capacity for ATP synthesis that kept various ATP-driven pumps active (i.e., energy-dependent sodium pump (Na +/K + - ATPases) and consequently limited the extend of lung graft tissue damages. Our study had nonetheless some limitations. First, the lung function was assessed during EVLP and we performed lung transplantation but we did not organize a longer post-transplantational follow-up period. Second, while the outbred Sprague Dawley rat strain selected for these experiments is suitable for EVLP experiments, it is less suitable for short-term transplantation settings and not suitable for survival experiments. We may use our transplantation model of the Brown Norway strain lungs tested during EVLP that are transplanted in the Lewis rat strain as a recipient for extended survival experiments [31]. Third, we did not use leucocyte filters during the rat EVLP which was documented to reduce the release of pro-inflammatory cytokines IL-6 in the perfusate and whose absence impairs the quality of the lung grafts [32]. This is the first study that directly compares a subnormothermic EVLP temperature in clinically relevant rat donor lungs that are subsequently transplanted in a short-term acute model. We showed that the 28 °C subnormothermic EVLP temperature have a strong beneficial potential for the physiological parameters and on the attenuation of I/R injury with a significantly reduced pro-inflammatory cytokine response when compared to normothermic temperature. Taking in account all results, this study suggests that subnormothermic EVLP temperature of 28 °C is a non-inferior setting in comparison to the clinically approved normothermic setting with a potential to reduce I/R injury. This hypothesis should be tested in a large animal model in order to obtain more stable and reliable assessments.

## Figures and Tables

**Table 1:** Perfusate, plasma and tissue cytokines, chemokines and mediators of wound healing and tissue repair in the study groups.

M (SD)	Perfusate	Perfusate	Plasma	Plasma	Tissue	Tissue
	Normo. (N = 8)	Subn. (N = 9)	Normo. (N = 6)	Subno. (N = 6)	Normo. (N = 6)	Subno. (N = 6)
TNF-α	1589 (1073) ***	59.65 (69.4)	1679 (1962)	633 (451.3)	140.4 (23.86) *	105.9 (26.96)
VEGF	0 (0)	0 (0)	1271 (1683)	744.2 (526.2)		
MCP-1	109.5 (38.65) ***	20.29 (9.96)	2761 (4942)	838.1 (382.5)		
GM-CSF	1.23 (0.90) *	0.50 (0.14)	562.2 (1062)	167.5 (113.7)		
RANTES	12.01 (6.26) *	6.15 (3.19)	157.2 (51.79)	278.3 (153.8)		
MIP3-α	6.31 (7.64) *	0.58 (0.02)	165.3 (69.47)	139 (89)		
MIP1-α	3018 (3064) *	372.9 (201.8)	713.5 (758.5)	287.1 (186.1)		
IFN-γ	0 (0)	0 (0)	352.8 (53.03)	464.7 (363.5)		
M-CSF	0.67 (0.39)	0.63 (0.53)	35.63 (8.41)	39.43 (29.79)		
G-CSF	0.18 (0.15) *	0.042 (0.00)	13.98 (1.87)	18.28 (18.67)		
GRO/KC	4536 (2450) ***	192.5 (206)	584.7(397.2)	407.6 (306.9)		
IL1-α	0.25 (0.33)	0.15 (0.14)	144.8 (50.02)	171.2 (99.19)		
IL1-β	9.65 (5.75)	11.27 (5.72)	1014 (1508)	423 (272.8)	489.5 (194.1) *	250.8 (165.2)
IL-2	0 (0)	0 (0)	1632 (264.9)	1810 (935.1)		
IL-4	1.20 (0.85)	0.60 (0.54)	142.5 (21.3)	180 (119.3)		
IL-5	6.05 (5.33)	4.92 (3.41)	159.3 (19.26)	166.3 (46.47)		
IL-6	134.7 (81.87)	3.032 (0.02)	826.7 (517.5)	607 (419.1)	612.5 (214.7)	394.1 (169.8)
IL-7	520.6 (929.7)	196.4 (138.9)	0.34 (0.34)	0.2 (0.002)		
IL-10	5.41 (6.11)	1.89 (3.04)	3569 (2739)	5886 (4518)		
IL-12 (p70)	1.851 (1.30)	1.036 (1.203)	191.8 (62.04)	226.3 (141.4)		
IL-13	0 (0)	0 (0)	67.67 (11.36)	94.81 (71.94)		
IL-17A	0.92 (0.22)	0.8 (0)	99.59 (10.04)	115.6 (61.64)		
IL-18	0 (0)	0 (0)	890 (222.8)	1167 (512.3)		

Cytokines, chemokines and mediators of wound healing and tissue repair in (pg/mL). Summary of the means (M) and the standard deviations (SD) for perfusate (after 4 h EVLP) or for the plasma and the lung tissue (after 2 h transplantation). Note * *p* ≤ 0.05; *** *p* ≤ 0.001; Normo: normothermic; Subno: subnormothermic.

## Data Availability

The data sets generated during and/or analyzed during the current study are available from the corresponding author on reasonable request.

## Abbreviations

Cdyn	dynamic compliance
DAMPS	damage-associated molecular patterns
EVLP	ex vivo lung perfusion
I/R	ischemia-reperfusion
MPO	myeloperoxidase
PAP	pulmonary arterial pressure
PEEP	positive end-expiratory pressure
PGD	primary graft dysfunction
PVR	pulmonary vascular resistance
ROS	reactive oxygen species
TPL	transplantation
